# Deciphering the Biological Significance of ADAR1–Z-RNA Interactions

**DOI:** 10.3390/ijms222111435

**Published:** 2021-10-23

**Authors:** Taisuke Nakahama, Yukio Kawahara

**Affiliations:** 1 Department of RNA Biology and Neuroscience, Graduate School of Medicine, Osaka University, Osaka 565-0871, Japan; nakahama@rna.med.osaka-u.ac.jp; 2 Integrated Frontier Research for Medical Science Division, Institute for Open and Transdisciplinary Research Initiatives (OTRI), Osaka University, Osaka 565-0871, Japan

**Keywords:** ADAR1, AGS, IGS, interferonopathy, MDA5, RNA editing, Z-RNA

## Abstract

Adenosine deaminase acting on RNA 1 (ADAR1) is an enzyme responsible for double-stranded RNA (dsRNA)-specific adenosine-to-inosine RNA editing, which is estimated to occur at over 100 million sites in humans. ADAR1 is composed of two isoforms transcribed from different promoters: p150 and N-terminal truncated p110. Deletion of ADAR1 p150 in mice activates melanoma differentiation-associated protein 5 (MDA5)-sensing pathway, which recognizes endogenous unedited RNA as non-self. In contrast, we have recently demonstrated that ADAR1 p110-mediated RNA editing does not contribute to this function, implying that a unique Z-DNA/RNA-binding domain α (Zα) in the N terminus of ADAR1 p150 provides specific RNA editing, which is critical for preventing MDA5 activation. In addition, a mutation in the Zα domain is identified in patients with Aicardi–Goutières syndrome (AGS), an inherited encephalopathy characterized by overproduction of type I interferon. Accordingly, we and other groups have recently demonstrated that *Adar1* Zα-mutated mice show MDA5-dependent type I interferon responses. Furthermore, one such mutant mouse carrying a W197A point mutation in the Zα domain, which inhibits Z-RNA binding, manifests AGS-like encephalopathy. These findings collectively suggest that Z-RNA binding by ADAR1 p150 is essential for proper RNA editing at certain sites, preventing aberrant MDA5 activation.

## 1. Introduction

**Figure 1:**
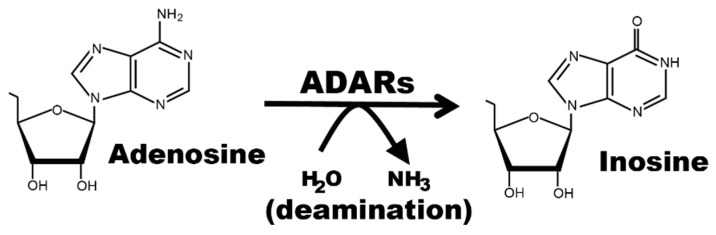
Adenosine-to-inosine RNA editing. Adenosine deaminases acting on RNA (ADARs) convert adenosine into inosine through a deamination reaction.

**Figure 2:**
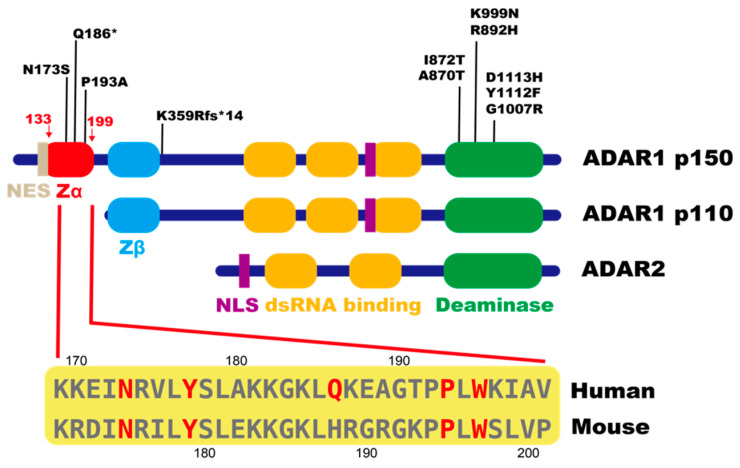
Structural representation of active human ADARs. Both ADAR1 and ADAR2 comprise double-stranded (ds)RNA-binding domains (orange), a C-terminal deaminase domain (green), and a nuclear localization signal (NLS; shown in purple). Both ADAR1 p150 and p110 comprise Z-DNA/RNA-binding domain β (Zβ; shown in light blue), which loses binding capacity to Z-DNA/RNA. In contrast, ADAR1 p150-specific Zα (red) can bind to Z-DNA/RNA. A nuclear export signal (NES; shown in light brown) is present only in the p150 isoform, which is predominantly localized in the cytoplasm. Amino acid substitution resulting from point mutations in the *ADAR1* gene, identified in patients with Aicardi–Goutières syndrome (AGS), is also shown. Amino acid sequences of a part of Zα in human and mouse ADAR 150 are shown below. Critical residues for Z-DNA/RNA binding and resides mutated in patients with AGS are shown in red.

Multiple lines of evidence have demonstrated that RNA modifications play a variety of roles in regulating the fate of each transcript, including recoding, splicing, mRNA decay, and translation. In addition, with the implementation of mRNA vaccines, the crucial roles of RNA modifications in distinguishing self and non-self RNAs have been highlighted [1]. Inosine is a modified nucleotide abundantly present in mRNA and is converted from adenosine via a deamination reaction, which is termed RNA editing [2,3,4] (Figure 1). This type of RNA editing is catalyzed by two active adenosine deaminases acting on the RNAs (ADARs) in mammals—ADAR1 and ADAR2 (Figure 2). Given that ADAR1 and ADAR2 commonly contain double-stranded RNA (dsRNA)-binding domains (dsRBDs), as well as a C-terminal deaminase domain, inosine is inserted into the dsRNA structure. Although it is extremely rare, inosine is found in certain coding regions, which form a dsRNA structure with the adjacent intron or within a single exon [5,6,7,8,9]. RNA editing in these coding regions can affect the amino acid sequence, altering the physiological function of the resultant protein, given that inosine is recognized as if it were guanosine by translational machinery. One such example is the Q/R site of *Gria2*, which encodes the GluA2 subunit of α-Amino-3-hydroxy-5-methyl-4-isoxazolepropionate (AMPA) type glutamate receptors. AMPA receptors are homo- or hetero-oligomeric assemblies composed of four subunits, GluA1, GluA2, GluA3, and GluA4, in various combinations. Among these subunits, only *Gria2* is edited solely by ADAR2 at the Q/R site almost completely in neurons, substituting the glutamine (Q) to the arginine (R) [6]. This modification affects the properties of the subunit, in which edited GluA2-containing receptors have low Ca^2+^ conductance, whereas unedited GluA2-containing receptors are Ca^2+^ permeable. However, most RNA-editing sites are found in repetitive elements, especially in short interspersed elements (SINEs), located in introns and 3′ untranslated regions (UTRs), given that two inverted repetitive elements form a long dsRNA structure. Therefore, the number of RNA-editing sites is determined by the abundance of repetitive elements [10,11,12,13,14,15,16]. In humans, at least 85% of precursor or mature mRNAs presumably form a dsRNA structure, and therefore, it is estimated that RNA editing occurs at more than 100 million sites, especially in *Alu* elements, the most abundant SINEs in humans [10,17,18]. This number is much greater than that in mice (~50 thousand sites), reflecting the volume of a repeat repertoire [15,16,19,20].

ADAR1 is expressed as two isoforms: longer p150 and short p110, which are transcribed from the same genomic loci using different promoters and share Z-DNA/RNA-binding domain β (Zβ), dsRBDs, and the deaminase domain [21] (Figure 2). In contrast to N-terminal-truncated ADAR1 p110, which is driven by a constitutive promoter, ADAR1 p150 contains a unique Zα in the N terminus and is controlled under an interferon (IFN)-inducible promoter [22,23]. Furthermore, ADAR1 p110 and ADAR2 are highly expressed in the brain and are mainly localized in the nucleus, especially in the nucleolus [24,25,26,27]. In contrast, ADAR1 p150 is expressed at very low levels in the mouse brain but highly expressed in lymphoid organs, such as the thymus and spleen [26,27]. In addition, ADAR1 p150 possesses a nuclear export signal (NES), which is partially overlapped with Zα (Figure 2). Therefore, it predominantly localizes in the cytoplasm but might shuttle between the nucleus and cytoplasm, especially under certain conditions, such as viral infections [21,28,29,30]. *Adar2* knockout (KO) mice die up to 3 weeks after birth due to progressive seizures [31]. This is caused by an absence of recoding at the Q/R site of *Gria2*. Therefore, the expression of edited *Gria2* rescues the lethality of *Adar2* KO mice [31,32]. In contrast, *Adar1* KO mice exhibit embryonic lethality at E11.5–12 with massive apoptosis and the excess expression of type I IFN [33,34,35], suggesting that the biological significance of ADAR1-mediated RNA editing is different from that of ADAR2. In addition, *Adar1 p150*-specific KO mice also manifest embryonic lethality at E11–12 [36], suggesting the contribution of ADAR1 p150 to normal development at early stages. However, many longstanding questions remain. For instance, what is the biological significance of RNA editing at repetitive elements? Why do *Adar1* KO mice exhibit embryonic lethality with elevated expression of type I IFN? How do functions differ between ADAR1 p110 and p150? What is the role of Zα, which is unique to ADAR1 p150? We have obtained some, though not all, answers to these questions. In this review, we introduce recent findings that offer crucial clues to such questions and discuss what remains unsolved.

## 2. ADAR1-Mediated RNA Editing Is Essential to Avoid MDA5 Sensing of Endogenous dsRNAs

Although the number is very limited, ADAR1 participates in RNA editing at coding sites [5,16]. For instance, RNA editing at five sites of serotonin 5-HT_2C_ receptor, which are catalyzed by both ADAR1 and ADAR2, affects the efficacy of G protein coupling [7]. Consequently, the pattern of RNA editing regulates energy metabolism and mood in mice [37,38,39,40]. The Q/R site of the kainite glutamate GluK2 subunit is also edited by both ADAR1 and ADAR2, which regulates Ca^2+^ permeability and synaptic plasticity [5,41]. However, the majority of coding sites are edited by ADAR2, and therefore, ADAR1-mediated protein recoding is not involved in embryonic lethality found in *Adar1* KO mice [16,42].

**Figure 3:**
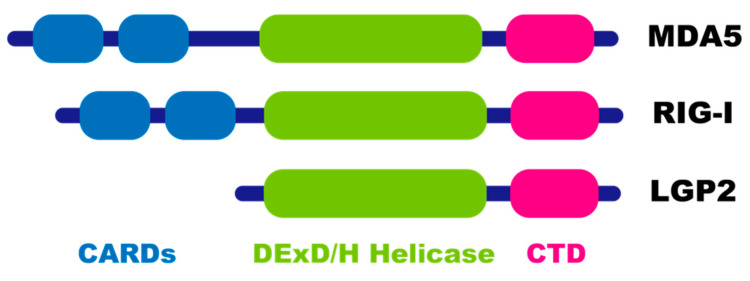
Structural representation of retinoic acid-inducible gene I (RIG-I)-like receptor family members. Melanoma differentiation-associated protein 5 (MDA5) and RIG-I comprise two caspase activation and recruitment domains (CARDs; shown in blue), which mediate signal transduction through interaction with the mitochondrial antiviral-signaling protein (MAVS), with a DExD/H-box RNA helicase domain (light green) and a C-terminal domain (CTD; shown in pink), both of which are required for RNA binding. LGP2 lacks CARDs.

In contrast, one of the great findings regarding the biological significance of ADAR1-mediated RNA editing is that embryonic lethality found in *Adar1* KO mice is rescued by concurrent deletion of either *Ifih1*-encoded melanoma differentiation-associated protein 5 (MDA5) or its downstream mitochondrial antiviral signaling protein (MAVS) [43,44]. In addition, deletion of either MDA5 or MAVS also ameliorates the elevated expression of IFN-stimulated genes (ISGs) found in *Adar1* KO mice. MDA5 belongs to the retinoic acid-inducible gene I (RIG-I)-like receptor (RLR) family, with RIG-I and LGP2 (Figure 3). LGP2 lacks caspase recruitment domains (CARDs), which are required for MAVS activation [45]. In contrast, MDA5 and RIG-I are cytosolic sensors for exogenous dsRNA and promote transcription of ISGs through MAVS. MDA5 recognizes longer dsRNAs, whereas RIG-I binds to dsRNAs containing the 5′ppp and blunt end [44,46,47,48,49]. Considering embryonic lethality is not rescued by concurrent deletion of RIG-I, and endogenous repeat elements can activate MDA5 [44,50], these findings indicate that ADAR1-mediated RNA editing prevents aberrant MDA5 recognition of endogenous dsRNA as non-self. Nevertheless, most *Adar1/Mavs* double KO (dKO) and *Adar1/Ifih1* dKO mice die just after birth and cannot survive beyond P10 [43,44]. In contrast, ~60% of *Adar1 p150/Mavs* dKO mice can survive more than 20 days after birth [44]. Furthermore, although *Adar1^E861A/E861A^* mice that express inactive ADAR1 with an E861A substitution in the deaminase domain exhibit embryonic lethality at E13.5 with an excess expression of ISGs, *Adar1^E861A/E861A^/Ifih1* KO mice survive until adulthood [5,42,51,52]. These findings collectively suggest that both ADAR1 p110 and p150 might have functions other than RNA editing, which is especially critical for postnatal early development.

## 3. ADAR1 p110 Is Dispensable for Blocking MDA5-Sensing Pathway

In order to investigate the contribution of ADAR1 p110 to the MDA5-sensing pathway, we created *Adar1 p110*-specific KO mice by deleting the constitutive promoter and ADAR1 p110-specific first exon [27]. This manipulation did not affect ADAR1 p150 expression. In addition, ADAR1 p110 expression is not altered in cells derived from *Adar1 p150*-specific KO mice [44]. These findings indicate that the expression of each ADAR1 isoform can be maintained by each isoform-specific promoter. Notably, *Adar1 p110*-specific KO mice show a high mortality rate during early postnatal days without an ISG signature [27]. This high mortality rate is not ameliorated by concurrent deletion of MDA5 but rescued by expressing inactive ADAR1 (E861A). These findings indicate that although causative abnormalities remain unspecified, ADAR1 p110 exerts RNA-editing-independent functions that are essential for early postnatal development. In addition, the high mortality rate found in *Adar1 p110*-specific KO mice explains, at least partly, the phenotypic differences between *Adar1/Mavs* dKO and *Adar1 p150/Mavs* dKO mice, and between *Adar1/Ifih1* dKO and *Adar1^E861A/E861A^/Ifih1* KO mice.

We further created *Adar1 p110/Adar2* dKO mice, in which increased expression of ISGs was not observed [27]. This is in contrast to the phenotypes found in *Adar1 p150*-specific KO mice, in which MDA5 is aberrantly activated [44]. Furthermore, the number of RNA-editing sites and editing frequency, especially in intronic regions, are dramatically reduced in *Adar1 p110/Adar2* dKO mice. In particular, substantial intronic editing is not detected in the brains of these mutant mice. Such findings indicate that RNA editing in intronic regions, which is catalyzed by nuclear ADAR1 p110 and ADAR2, is dispensable to prevent MDA5 activation. In contrast, although the number is fewer than 40 sites, RNA editing is observed in the 3′UTR of mRNA in the brains of *Adar1 p110/Adar2* dKO mice. These sites are definitely catalyzed by cytoplasmic ADAR1 p150, although its expression is extremely low [5,26,27,52]. Indeed, RNA editing in the 3′UTR of certain genes is well preserved in the absence of ADAR1 p110 and ADAR2 [27]. Collectively, these lines of evidence suggest the presence of ADAR1 p150-specific RNA-editing sites, which are essential to suppress aberrant MDA5 sensing of endogenous dsRNAs.

## 4. Mutations in Zα and the Deaminase Domain Are Associated with Aicardi–Goutières Syndrome

**Figure 4:**
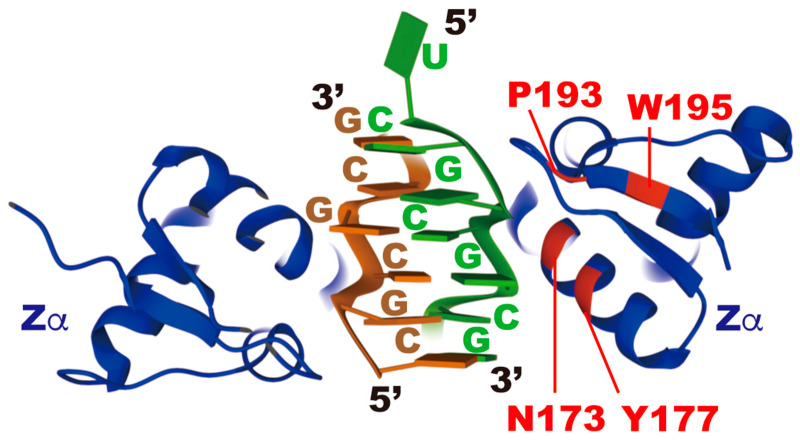
Crystal structure of Z-RNA bound to Zα of ADAR1 p150. Two Zα domains in blue bind to Z-RNA composed of dUr(CG)_3_ duplex in brown and green (PDB ID: 2GXB) [59]. Critical residues for Z-DNA/RNA binding (N173 and Y177 in the α helix, and W195 in the β sheet) and residues mutated in patients with AGS (N173 and P193) are shown in red.

Aicardi–Goutières syndrome (AGS) is a rare congenital interferonopathy with encephalopathy characterized by leukodystrophy and intracranial calcification, leading to brain atrophy [53]. In addition, patients manifest inflammatory symptoms in various organs, including liver, lung, and skin, accompanied by the overproduction of type I IFN. Symptom onset occurs during the perinatal stage, and most patients die within 10 years after birth [53,54]. To date, seven causative genes have been identified, including *ADAR1* (AGS type 6) and *IFIH1* (AGS type 7) [53,55,56,57]. In cases of AGS type 7, gain-of-function mutations make MDA5 more prone to be activated by reducing ligand specificity. In contrast, in cases of AGS type 6, most mutations are identified in the deaminase domain, which likely reduces RNA-editing activity [43,55] (Figure 2). Notably, although homozygous mutations have not been identified to date, a heterozygous point mutation exists at the position of amino acids 173 and 193 in ADAR1 p150-specific Zα, which converts asparagine 173 to serine (N173S), and proline to alanine (P193A), respectively [55,58] (Figure 2 and Figure 4). These mutations are accompanied by another mutation in the deaminase domain or one that leads to the loss of ADAR1 p150 expression. These findings suggest that Zα most likely plays a pivotal role in ADAR1 p150-specific regulation of RNA editing.

## 5. Zα Contributes to ADAR1 p150-Mediated RNA Editing

How does ADAR1 p150 regulate its specific RNA editing? Given that Zβ, dsRBDs, and the deaminase domain are shared between ADAR1 p110 and p150, differences between the two include the intracellular localization and the presence of Zα in the N terminus of the p150 isoform (Figure 2). Although ADAR1 p150 is predominantly localized in the cytoplasm, it might shuttle between the nucleus and cytoplasm [21,28,29,30]. However, substantial intronic editing is not detected in the brains of *Adar1 p110/Adar2* dKO mice, whereas RNA editing in the 3′UTR of certain mRNAs is preserved [27]. This finding suggests that ADAR1 p150-mediated RNA editing is generally executed in the cytoplasm, at least under normal conditions. In addition, ADAR1 p150 translocates into cytoplasmic stress granules (SGs) under abnormal conditions, such as viral infection and IFN-induced stress [60,61,62,63,64]. This translocation requires Zα and might sequester specific RNAs into SGs to escape MDA5 sensing or achieve efficient RNA editing. However, this translocation is not observed under normal conditions. Collectively, considering that ADAR1 p110 and p150 harbor the same deaminase domain, and ADAR1 p110 meets target RNAs in the nucleus prior to ADAR1 p150, the difference in intracellular localization cannot account for all ADAR1 p150-specific regulation of RNA editing.

Zα of ADAR1 p150 was originally identified as the domain that binds to left-handed Z-DNA composed of CG repeats [65,66,67,68]. However, ADAR1 p150 is an RNA-editing enzyme, predominantly localized in the cytoplasm. Therefore, although binding to Z-DNA may play a biological function under certain conditions [69], it is reasonable to postulate that this domain plays a role in RNA regulation. Indeed, Zα of ADAR1 p150 efficiently binds to Z-RNA composed of CG repeats [59,70,71] (Figure 4). Additionally, although in vivo Z-RNA sequences remain unknown, the addition of CG repeats to dsRNA increases ADAR1 p150-mediated RNA editing in vitro, which suggests Z-RNA formation affects the efficiency of RNA editing [72]. The periodic sequence composed of CG repeats in Z-RNA produces a zigzag in the course of the line that links alternating phosphates and therefore needs 12.4 bp per turn, which is in contrast to ~11 bp per turn in right-handed A-RNA [73,74]. The formation of Z-RNA requires higher energy, and therefore, this configuration is unstable in the absence of Zα. Three conserved residues—N173 and tyrosine 177 (Y177) in the α helix, and tryptophan 195 (W195) in the β sheet of Zα of human ADAR1 p150—play central roles in the interaction with Z-DNA, which is also applicable to Z-RNA [59,75] (Figure 2 and Figure 4). In particular, compared with N173A and Y177A substitution, W195A substitution is the most deleterious and results in the complete loses of binding to Z-DNA [76]. Therefore, we compared RNA-editing activity between wild-type ADAR1 p150 (WT) and ADAR1 p150 (W197A) by expressing the corresponding gene in mouse *Adar1/Adar2* dKO Raw 264.7 cells. W197 in mouse ADAR1 p150 corresponds to W195 in the human equivalent (Figure 2). Although cytoplasm-dominant localization and expression level are not affected by W197A substitution, the RNA-editing activity of ADAR1 p150 (W197A) is significantly lower than that of the wild-type at selective sites [77]. Furthermore, de Reuver et al. inserted Y177A with/without N173A mutations into ADAR1 p150 in human HEK293 cells and compared RNA-editing activity between wild-type ADAR1 p150 (WT) and Zα-mutated ADAR1 p150 after increasing the amount of ADAR1 p150 by the addition of IFN-α2 [78]. This analysis demonstrates that although the number of RNA-editing sites, including ADAR1 p110- and ADAR2-responsible sites, is not largely different, RNA editing in the 3′UTR seems more affected than in other regions. In addition, Zα-mutated ADAR1 p150-expressing HEK293 cells are more sensitive to the increased amount of MDA5 than wild-type cells [78]. Notably, the expression of dsRBD-mutated ADAR1 p150, in which Zα is intact, cannot edit any sites in *Adar1/Adar2* dKO Raw 264.7 cells, suggesting that the presence of Zα alone is not sufficient to induce RNA editing [77]. Collectively, these findings indicate that Z-RNA binding of ADAR1 p150 through the Zα domain is required for efficient RNA editing at certain sites, at least in vitro.

## 6. Preventing Z-RNA Binding of ADAR1 p150 Induces AGS-Like Encephalopathy

To elucidate the biological significance of Z-RNA-binding to ADAR1 p150 in vivo, various studies, analyzing mutant mice harboring single or double point mutation(s) in Zα, have been reported [77,78,79,80,81]. *Adar1* knock-in (KI) mice that harbor a P195A point mutation, corresponding to human AGS-causative P193A mutation (Figure 2), show no overt phenotypes [80,81]. However, *Adar1^P195A/-^* and *Adar1^P195A/p150−^* mice cannot survive beyond 3 and 4 months, respectively, with abnormalities in the spleen, kidney, and liver, and increased expression of ISGs, which is in contrast to the normal phenotypes of *Adar1*^+/−^ and *Adar1 p150*^+/−^ mice [80]. These findings suggest that the P193A mutation most likely affects the RNA-editing activity of ADAR1 p150 to some extent but not enough to induce phenotypic abnormalities, which might explain the reason why homozygous point mutations in Zα have not been identified in patients with AGS to date. In contrast, *Adar1* KI mice harboring N175A/Y179A point mutations exhibit increased expression of ISGs in multiple organs, which lasts more than a year [78,79]. N175A/Y179A point mutations correspond to an N173A/Y177A substitution in human ADAR1 p150, both of which reduce Z-DNA-binding capacity in vitro [76] (Figure 2 and Figure 4). However, although only male *Adar1^N175A:Y179A/N175A:Y179A^* mice are leaner, these mutant mice survive beyond a year without displaying specific abnormalities in certain organs, suggesting chronically enhanced expression of ISGs is not sufficient to induce abnormal pathology [78,79]. Notably, *Adar1 ^N175A:Y179A/−^* mice cannot survive beyond postnatal day 1, which is more deleterious than found for *Adar1^P195A/−^* mice [78,80]. This difference most likely reflects the remaining capacity of Z-RNA binding.

We created *Adar1* KI mice harboring either W197A or N175A/W197A point mutations, both of which result in a normal appearance at birth; however, such mice have severe growth retardation, and therefore, half of the mutant mice die within 6 weeks after birth [77]. The expression of ISGs is elevated in multiple organs, especially in the brains of *Adar1^W197A/W197A^* mice. In addition, the numbers of thymocytes and splenocytes were severely reduced and accompanied by atrophy of the thymus and spleen. Furthermore, differentiation into lineage marker (L)^−^/c-Kit (K)^+^/Sca-I (S)^−^ (LKS^−^) hematopoietic stem cells from LKS^+^ cells was impaired in the bone marrow of *Adar1^W197A/W197A^* mice, as similarly found in *Adar1* KO and *Adar1^E861A/E861A^* mice [34,51,77]. Brain atrophy with white matter vacuolation is characteristically observed in *Adar1^W197A/W197A^* mice, which is reminiscent of the encephalopathy found in patients with AGS. In addition, astrocytes and microglia are aberrantly activated without infiltration of lymphocytes, which provides a clue in elucidating mechanisms underlying the formation of leukodystrophy in patients with AGS. It should be noted that such phenotypic abnormalities and enhanced expression of ISGs are ameliorated by concurrent deletion of MDA5.

**Figure 5:**
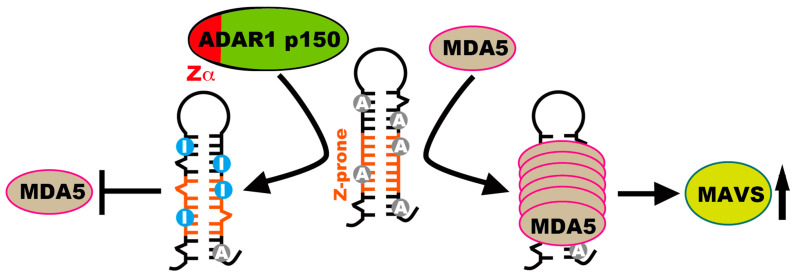
Preventing MDA5 sensing of dsRNAs by ADAR1 p150-mediated RNA editing. ADAR1 p150 binds to Z-prone sequences in dsRNAs through Zα, converting to Z-RNA and promoting adenosine (A)-to-inosine (I) RNA editing of dsRNAs. ADAR1 p150-specific RNA editing is required for prevention of sensing unedited dsRNAs by MDA5, leading to filament assembly and activating MAVS.

In *Adar1^N175A:Y179A/N175A:Y179A^* and *Adar1^W197A/W197A^* mice, the expression of ADAR1 p150, which is an ISG, is increased, leading to enhanced RNA editing at a subset of, but not all, target sites [77,79]. In addition, ADAR1 p110 and ADAR2 are normally expressed, which makes it difficult to understand the pattern of RNA editing in Zα-mutated *Adar1* KI mice. However, the phenotypes of *Adar1^W197A/E861A^* mice are more deleterious than those of *Adar1^W197A/W197A^* mice [77]. These findings suggest that reduced RNA editing at certain sites, which cannot be ameliorated by increased expression of mutant ADAR1 p150, causes aberrant activation of the MDA5-sensing pathway in these Zα-mutated *Adar1* KI mice [77,78,79] (Figure 5). Indeed, we found that some sites in the 3′UTR of Rnf168 and 2900026A02Rik show reduced RNA editing and do not respond to increased expression of ADAR1 p150 (W197A) in the brains of *Adar1^W197A/W197A^* mice [77]. Therefore, these sites most likely require Z-RNA binding of ADAR1 p150 through Zα to maintain the proper RNA-editing level (Figure 5).

## 7. Downstream of MDA5-Sensing Pathway Contributes to Mutated ADAR1 p150-Driven Pathology

Phenotypic and pathological abnormalities found in *Adar1^P195A/p150−^* and *Adar1^W197A/W197A^* mice are normalized by concurrent deletion of MDA5, which ameliorates the expression level of ISGs [77,80]. In addition, early postnatal lethality found in *Adar1 ^N175A:Y179A/−^* mice is rescued by concurrent deletion of MAVS [78]. These findings indicate that MDA5 is an initial sensor of unedited dsRNAs, which transmits signals to MAVS (Figure 5). However, elucidating downstream of the MDA5/MAVS axis has been hampered so far, given that concurrent deletion of STAT1, IFNα/β receptor subunit 1 (IFNAR1), IFNγ receptor (IFNGR), STING, RIG-I encoded by *Ddx58*, IFN regulatory factor 3 (IRF3), and protein kinase R (PKR) encoded by *Eif2ak2* all fail to rescue embryonic lethality found in *Adar1* KO mice, which is caused by a mixture of RNA-editing-dependent and-independent mechanisms [27,35,43,44,82,83]. In contrast, the expression of ADAR1 p110 is intact in *Adar1^P195A/p150−^* mice. In addition, pathological abnormalities and elevated expression of ISGs found in *Adar1^P195A/p150−^* mice are normalized by concurrent deletion of MDA5 [80], which suggests that the RNA-editing-independent function of ADAR1 150 has a minimal effect in these mutant mice if present. Therefore, Maurano et al. investigated downstream of the MDA5/MAVS axis by crossing *Adar1^P195A/p150−^* mice with other mutant mice in which candidate genes, such as *Dhx58* (encoding LGP2), *Ifnar1*, *Rnasel*, and *Eif2ak2*, are deleted [80]. Intriguingly, LGP2 deficiency completely rescued the abnormalities found in *Adar1^P195A/p150−^* mice, including a high mortality rate and elevated expression of ISGs. Although LGP2 lacks CARD, which is required for MAVS activation [45] (Figure 3), it interacts with MDA5, facilitating MDA5 fiber assembly on dsRNAs [84,85,86]. Therefore, LGP2 likely plays a supportive role in MDA5 sensing of unedited dsRNAs and transmitting signals to MAVS, which needs further investigation. Concurrent deletion of IFNAR1 extends survival of *Adar1* KO embryos by 3–4 days and partially ameliorates the enhanced expression of ISGs [43]. Therefore, as expected, the absence of the type I IFN receptor also completely rescues the abnormalities found in *Adar1^P195A/p150−^* mice [80]. These findings suggest that the type I IFN produced is downstream of MDA5 activation in a subset of cells, and is secreted to affect a much wider range of cells.

Oligoadenylate synthetase (OAS) proteins produce 2′,5′-oligoadenylate upon recognition of dsRNAs, which activates RNase L and eventually induces translational inhibition and apoptosis [87,88]. The lethal phenotype of ADAR1-deficient A549 cells is rescued by concurrent deletion of RNase L [89], which suggests the OAS-RNase L pathway as being downstream of the MDA5-sensing pathway or an alternative one. In addition, PKR is induced by IFN and activated upon recognition of dsRNAs, inhibiting translation and inducing the expression of genes associated with the integrated stress response. In normal and cancer cells, ADAR1 deficiency activates the PKR pathway, leading to translational shutdown and cell death or growth arrest [90,91,92,93,94]. However, the relationship between MDA5 and PKR pathways has not been clear by using cell culture models. Maurano et al. reported that RNase L deficiency cannot rescue the abnormalities found in *Adar1^P195A/p150−^* mice, indicating that the OAS–RNase L pathway is not likely involved in the sensing of unedited dsRNAs in vivo. In contrast, PKR deficiency completely rescues mortality and restores the weight loss found in *Adar1^P195A/p150−^* mice, although the expression of ISGs remains elevated [80]. These findings indicate that the PKR pathway that contributes to the formation of an abnormal pathology is downstream of the MDA5-sensing pathway. It should be noted that the phenotypes of *Adar1^W197A/W197A^* and *Adar1^W197A/E861A^* mice are severer than those found in *Adar1^P195A/p150−^* mice [77,80]. This difference is attributable to the remaining capacity for Z-RNA binding, which is definitely required for ADAR1 p150-mediated RNA editing at certain critical sites. Therefore, the extent of the recovery of abnormal phenotypes and pathology found in *Adar1^W197A/W197A^* mice by the concurrent deletion of IFNAR1 and PKR merits investigation.

## 8. Unsolved Issues and Future Challenges Regarding Z-RNA and ADAR1 p150 Interactions

During the past decade, various aspects of ADAR1-mediated RNA editing have been elucidated. However, many unsolved issues remain. First, where Z-RNA is embedded in the dsRNA structure remains unknown. Given that the dsRNA structure in vivo is largely incomplete, it is more difficult to search for potential Z-RNA sequences compared with DNA. Recent studies revealed that primate-specific *Alu* elements contain CG-rich sequences, which can form Z-RNA [67,95]. Nichols et al. proposed that ADAR1 p150 binds to the Z-prone sequence, destabilizing neighboring right-handed A-form regions, and subsequent binding of another ADAR1 p150 stabilizes a Z-conformation surrounded by A–Z junctions [67,95]. Although this information offers an important clue, such CG-rich sequences are not found in rodent SINEs. However, we are now aware of the existence of ADAR1 p150-specific sites and that aberrant MDA5 activation is suppressed by only ADAR1 p150-mediated RNA editing (Figure 5). Therefore, a comparison of preferential editing sites between ADAR1 p110 and p150 may shed light on Z-RNA sequences recognized by Zα in vivo.

Second, how the insertion of inosine into dsRNAs can block MDA5 sensing remains unknown. The increasing amount of MDA5 forcibly expressed induces strong activation of an IFNβ reporter in wild-type HEK293 cells; this is enhanced in cells expressing Zα-mutated ADAR1 p150 [78]. ADAR1 p150 is expressed at the lowest level in the mouse brain where the MDA5 expression level is also low, in contrast to the high expression of ADAR1 p150 and MDA5 in the thymus [27]. Therefore, a proper RNA-editing level seems to be determined by the expression level of MDA5, regardless of the amount of dsRNAs, at least in part. Although the reason why ADAR1 p150 is expressed at the lowest level in the mouse brain remains unknown, the most enhanced expression of ISGs in the brains of *Adar1^W197A/W197A^* mice might be attributed to a lack of extra capacity of RNA editing in the brain. A–U base pairs are destabilized by RNA editing. However, ADARs prefer A–C mismatches [96,97], which are stabilized by RNA editing. Therefore, it is difficult to predict how a combination of inosine insertion, which depends upon the expression of ADAR1 p150 and target mRNAs, alters each dsRNA structure [51,98,99]. In addition, the identification of endogenous RNAs bound to MDA5 is challenging, given that MDA5 forms helical filaments for binding to dsRNAs [50]. Since the number of ADAR p150-mediated RNA-editing sites is very limited in the mouse brain, clues are expected from the identification of sites highly edited by ADAR1 150 in such organs [27].

Finally, RNA-editing-independent functions of ADAR1 p150, in addition to that of ADAR1 p110, merits investigation. An ADAR1 p110 deficiency in mice causes early postnatal death in an RNA-editing-independent manner, which is not specified [27]. In addition, ~40% of *Adar1 p150/Mavs* dKO mice cannot survive more than 20 days after birth, in contrast to the long-term survival of *Adar1^E861A/E861A^/Ifih1* KO mice [44,52]. Notably, *Adar1^Δ7–9^* mutant mice, in which exon 7–9 of *Adar1* is deleted, show milder phenotypes, compared with *Adar1* KO mice, in which exon 2–13 is deleted [33,83]. In *Adar1^Δ7–9^* mutant mice, truncated ADAR1 p150 and p110 are expressed, which lose the third dsRBD, including a nuclear localization signal, and editing activity (Figure 2). Therefore, although localization of truncated ADAR1 p110 is perturbed, cytoplasmic localization of truncated ADAR1 p150 is preserved, which might contribute to milder phenotypes. Collectively, the possibility remains that ADAR1 150 might have an RNA-editing-independent function by binding to dsRNAs through Zα with dsRBDs.

## 9. Conclusions

In this review, we summarized many important findings regarding ADAR1-mediated RNA editing and its physiological relevance. We now understand that ADAR1 exerts RNA-editing-dependent and -independent functions and that the targets of p110 and p150 isoforms are not necessarily identical. In particular, aberrant MDA5 recognition of endogenous dsRNAs is prevented by only ADAR1 p150-mediated RNA editing, in which Z-RNA recognition through Zα is indispensable. *Adar1^W197A/W197A^* mice, in which Zα loses the binding capacity for Z-RNA, manifest encephalopathy with gliosis, reminiscent of AGS. Collectively, ADAR1 p150–Z-RNA interactions are essential for maintaining proper RNA editing at certain sites, as well as for cellular homeostasis. The mechanisms underlying the escape of MDA5 sensing via RNA editing remain unclear. In addition, preferential sequences for forming Z-RNA in vivo need further investigation.

## Data Availability

Not applicable.
